# Effects of sugammadex versus neostigmine on postoperative nausea and vomiting after general anesthesia in adult patients:a single-center retrospective study

**DOI:** 10.1038/s41598-023-32730-1

**Published:** 2023-04-03

**Authors:** Jae-Woo Ju, In Eob Hwang, Hye-Yeon Cho, Seong Mi Yang, Won Ho Kim, Ho-Jin Lee

**Affiliations:** 1 Department of Anesthesiology and Pain Medicine, Seoul National University Hospital, 101 Daehak-ro, Jongno-gu, Seoul, 03080 Republic of Korea; 2 Department of Anesthesiology and Pain Medicine, Seoul National University College of Medicine, Seoul, Republic of Korea; 3 Department of Anesthesiology and Pain Medicine, Asan Medical Center, Seoul, Republic of Korea

**Keywords:** Disease prevention, Translational research, Surgery

## Abstract

We aimed to compare the effect of sugammadex to that of neostigmine with respect to the occurrence of postoperative nausea and vomiting (PONV) during the first 24 h following general anesthesia. This retrospective cohort study included patients who underwent elective surgery under general anesthesia in 2020 at an academic medical center in Seoul, South Korea. The exposure groups were determined according to whether the patient received sugammadex or neostigmine as a reversal agent. The primary outcome was PONV occurrence during the first 24 h postoperatively (overall). The association between the type of reversal agent and primary outcome was investigated using logistic regression while adjusting for confounding variables using stabilized inverse probability of treatment weighting (sIPTW). Of the 10,912 patients included in this study, 5,918 (54.2%) received sugammadex. Sugammadex was associated with a significantly lower incidence of overall PONV (15.8% vs. 17.7%; odds ratio, 0.87; 95% confidence interval [CI], 0.79–0.97; *P* = 0.010) after sIPTW. In conclusion, compared with neostigmine/glycopyrrolate, sugammadex use has a lower risk of PONV during the first 24 h following general anesthesia.

## Introduction

Postoperative nausea and vomiting (PONV) after general anesthesia occurs in 20–30% of patients^1^. This complication could cause patient discomfort, delayed resumption of postoperative oral intake, delayed discharge, and increased medical costs^2,3^. Efforts have been made to prevent PONV^4^, but complete prevention has thus far failed. The recent guidelines for PONV management have proposed using sugammadex for neuromuscular blockade (NMB) reversal as a strategy to reduce the baseline risk of PONV^4^. This recommendation was based on a recent meta-analysis that compared sugammadex with neostigmine and found a significantly lower incidence of PONV after treatment with sugammadex (odds ratio [OR]: 0.52; 95% confidence interval [CI], 0.28–0.97, *P* = 0.04)^5^. A large population-based multicenter retrospective study comparing sugammadex with neostigmine also reported a similar reduction in PONV incidence after treatment with sugammadex, before and after propensity-score matching^6^.

However, all six randomized-controlled trials (RCTs) analyzed in the aforementioned meta-analysis monitored PONV only during the early postoperative period (until discharge from the recovery room)^5^ and did not cover the first 24-h postoperatively, which corresponds to the minimum observation period recommended^7^. In addition, the effect of neostigmine on PONV has been controversial^8^; therefore, we have cautiously cast doubt on whether using sugammadex instead of neostigmine could be an effective strategy to reduce the occurrence of PONV during the first 24 h postoperatively^4^. Moreover, of these six RCTs, only one small RCT was conducted under propofol-based total intravenous anesthesia (TIVA), and this trial found no advantage of sugammadex over neostigmine^9^. Similarly, most patients (95.6%) in the retrospective study received inhalation anesthesia^6^. In general, there is a paucity of data on the impact of the general anesthesia type (inhalation anesthesia vs. propofol-based TIVA) on the association between sugammadex use and PONV occurrence.

The primary objective of this retrospective study was to investigate the association between the type of reversal agent (sugammadex vs. neostigmine) and PONV occurrence during the first 24 h postoperatively after elective surgery under general anesthesia in adult patients. We hypothesized that sugammadex use would not be significantly associated with the occurrence of PONV during the first 24 h postoperatively. The secondary objective was to investigate whether the type of general anesthesia affected the association between sugammadex use and the occurrence of PONV during the first 24 h postoperatively.

## Results

**Figure 1:**
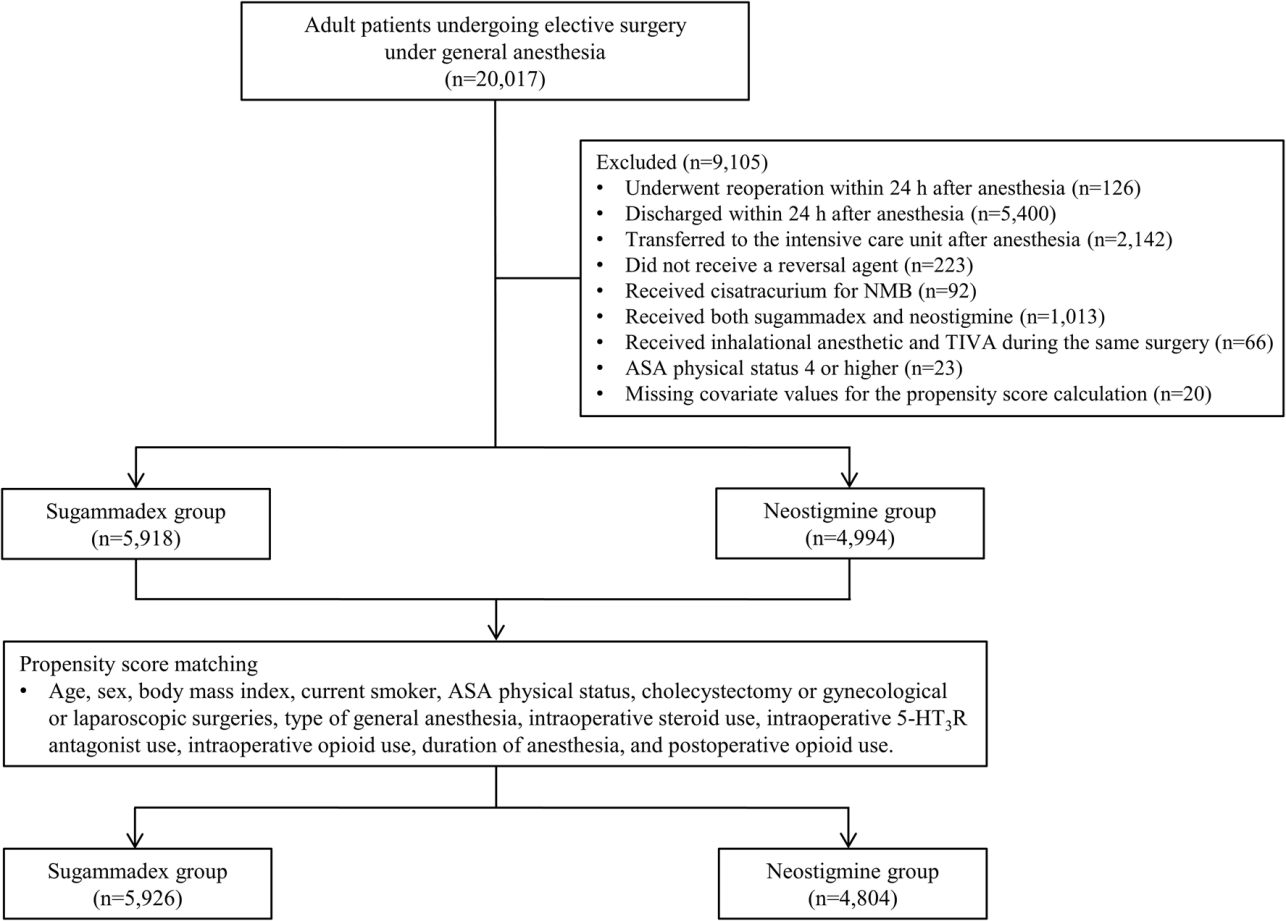
Flow diagram of the study. ASA, American Society of Anesthesiologists; NMB, neuromuscular blockade; TIVA, total intravenous anesthesia; 5-HT_3_R, 5-hydroxytryptamine receptor.

Of the 20,017 adult patients who underwent elective surgery under general anesthesia during the study period, 10,912 patients were eligible and included in the analysis (Fig. 1). Among them, 5,918 (54.2%) received sugammadex and 4,994 (45.8%) received neostigmine and glycopyrrolate for NMB reversal. Table 1 presents the between-group comparison of baseline characteristics before and after stabilized inverse probability of treatment weighting (sIPTW). There were significant differences in age, sex, American Society of Anesthesiologists (ASA) physical status score, cholecystectomy or gynecological or laparoscopic surgery, type of general anesthesia, use of intraoperative steroids and 5-HT_3_R antagonists, duration of anesthesia, and postoperative opioid use before performing sIPTW (standardized mean difference [SMD] > 0.1) but not after, except for sex, for which the SMD was acceptable (SMD = 0.101).

**Table 1:** Comparison of baseline characteristics and perioperative parameters between patients treated with sugammadex and with neostigmine before and after adjusting for confounding by sIPTW in the total cohort.

Characteristics	Before sIPTW			After sIPTW^a^		
	Sugammadex (n = 5918)	Neostigmine (n = 4994)	SMD	Sugammadex (n = 5928)	Neostigmine (n = 4736)	SMD
Age, years	61 (49–69)	54 (42–64)	0.363	59 (46–68)	57 (45–67)	0.070
Female	3047 (51.5)	3527 (70.6)	0.400	3531.7 (59.6)	3053.4 (64.5)	0.101
Body mass index, kg·m^−2^	23.9 (21.8–26.3)	23.8(21.6–26.3)	0.025	23.8 (21.6–26.3)	23.8 (21.6–26.2)	0.024
Current smoker	435 (7.4)	328 (6.6)	0.031	434.5 (7.3)	318.7 (6.7)	0.023
History of PONV	151 (2.6)	104 (2.1)	0.031	150.3 (2.5)	121 (2.6)	0.001
ASA physical status I/II/III	752 (12.7)/4505 (76.1)/661 (11.2)	913 (18.3)/3773 (74.7)/348 (7.0)	0.200	918 (15.5)/4422 (74.6)/589 (9.9)	761 (16.1)/3534 (74.6)/441 (9.3)	0.025
Cholecystectomy or gynecological or laparoscopic surgeries	2733 (46.2)	800 (16.0)	0.689	1934 (32.6)	1552 (32.8)	0.003
Extent of surgery, minor/intermediate/major	487 (8.2)/ 384 (6.5)/ 5047 (85.3)	1303 (26.1)/ 1225 (24.5)/ 2466 (49.4)	0.830	1055 (17.8)/ 861 (14.5)/ 4012 (67.7)	856 (18.1)/ 746 (15.7)/ 3134 (66.2)	0.037
Type of general anesthesia			0.321			0.027
Total intravenous anesthesia	1446 (24.4)	1958 (39.2)		1927 (32.5)	1600 (33.8)	
Inhalation anesthesia	4472 (75.6)	3036 (61.1)		3902 (67.5)	3136 (66.2)	
Intraoperative steroid use	2994 (50.6)	965 (19.3)	0.694	2066 (34.9)	1500 (31.7)	0.068
Intraoperative 5-HT_3_R antagonist use	5537 (93.6)	3847 (77.0)	0.480	5198 (87.7)	4029 (85.1)	0.076
Intraoperative opioid use	5902 (99.7)	4940 (98.9)	0.099	5887 (99.3)	4704 (99.3)	0.004
Duration of anesthesia, hour	2.65 (1.93–3.83)	2.40 (1.67–3.50)	0.172	2.43 (1.65–3.60)	2.55 (1.77–3.58)	0.025
Postoperative opioid use^b^	5208 (88)	3230 (64.7)	0.571	4517 (76.2)	3552 (75)	0.028

The values are presented as the medians (interquartile range) or as the numbers (%).

^a^Stabilized weights are used to adjust for confounding. The following variables are used as contributors to the propensity score: age, sex, body mass index, current smoker, ASA physical status, cholecystectomy or gynecological or laparoscopic surgeries, type of general anesthesia, intraoperative steroid use, intraoperative 5-HT_3_R antagonist use, intraoperative opioid use, and duration of anesthesia. Postoperative opioid use is not used for propensity score calculation.

^b^During the first 24 h postoperatively.

ASA, American Society of Anesthesiologists; SMD, standardized mean difference; sIPTW, stabilized inverse probability of treatment weighting; 5-HT_3_R, 5-hydroxytryptamine receptor.

Table 2 presents the ORs of using sugammadex for the primary and secondary outcomes before and after sIPTW. Patients who received sugammadex had a significantly lower rate of overall and early PONV than those who received neostigmine before sIPTW. After sIPTW, sugammadex (vs. neostigmine) was associated with a significantly lower rate of overall PONV (15.8% vs. 17.7%, respectively; OR, 0.87; 95% CI, 0.79–0.97; *P* = 0.010). Sugammadex (vs. neostigmine) was also associated with a significantly lower rate of early PONV (7.6% vs. 9.7%, OR, 0.77; 98.3% CI, 0.65–0.91; *P* < 0.001) and antiemetic use within the first 24 h postoperatively (11.8% vs. 14.3%, OR, 0.80; 98.3% CI, 0.70–0.92; *P* < 0.001) after sIPTW.

**Table 2:** Comparison of probability of primary and secondary outcomes between sugammadex use and neostigmine use, before and after adjusting for confounding by sIPTW, in the total cohort.

Primary outcome	Before sIPTW				After sIPTW^a^			
	Sugammadex (n = 5918)	Neostigmine (n = 4994)	Odds ratio^b^ (95% CI)	*P* value	Sugammadex (n = 5928)	Neostigmine (n = 4736)	Odds ratio^b^ (95% CI)	*P* value
Overall PONV (within 24 h)	964 (16.3)	898 (18.0)	0.89 (0.80–0.98)	0.019	936 (15.8)	837 (17.7)	0.87 (0.79–0.97)	0.010

Secondary outcome	Sugammadex (n = 5918)	Neostigmine (n = 4994)	Odds ratio^b^ (98.3% CI)	*P* value^c^	Sugammadex (n = 5928)	Neostigmine (n = 4736)	Odds ratio^b^ (98.3% CI)	*P* value^c^
Early PONV (0–2 h)	427 (7.2)	499 (10)	0.70 (0.59–0.83)	< 0.001	453 (7.6)	460 (9.7)	0.77 (0.65–0.91)	< 0.001
Delayed PONV (2–24 h)	687 (11.6)	592 (11.9)	0.98 (0.85–1.13)	0.691	655 (11)	563 (11.9)	0.92 (0.80–1.06)	0.171
Antiemetic use (within 24 h)	732 (12.4)	680 (13.6)	0.90 (0.78–1.03)	0.053	700 (11.8)	679 (14.3)	0.80 (0.70–0.92)	< 0.001

*CI* Confidence interval, *PONV* Postoperative nausea and vomiting, sIPTW, stabilized inverse probability of treatment weighting.

^a^Stabilized weights are used to adjust for confounding.

^b^Odds ratios estimate the probability of the given outcome in patients who received sugammadex versus patients who received neostigmine for each outcome.

^c^Statistical significance corrected by the Bonferroni correction to adjust for increased type I error by multiple testing (*P* < 0.05/3 = 0.017).

Interaction analysis revealed that there were no significant interactions among sugammadex use and type of general anesthesia for the primary and secondary outcomes except antiemetic use during the first 24 h postoperatively (Table 3). Multivariable logistic regression analysis showed that sugammadex use was significantly associated with overall and early PONV occurrence (overall: OR, 0.87; 95% CI, 0.77–0.98; *P* = 0.023; early: OR, 0.81; 95% CI, 0.68–0.96; *P* = 0.013; Table 4). Given that there were no significant interactions among sugammadex use and type of general anesthesia for the overall and early PONV occurrence, their interaction term was not included in these models.

**Table 3:** Interaction of primary and secondary outcomes between the use of sugammadex (vs neostigmine) and total intravenous anesthesia (vs inhalation anesthesia).

	Interaction* P* value
Primary outcome	
Overall PONV (within 24 h)	0.966
Secondary outcome	
Early PONV (0–2 h)	0.984
Delayed PONV (2–24 h)	0.502
Antiemetic use (within 24 h)	0.036

*PONV* postoperative nausea and vomiting.

**Table 4:** Predictive factors associated with postoperative nausea and vomiting during the first and 24 h after general anesthesia (GA) based on binary multivariable logistic regression analyses.

	During the first 2 h after GA		During the first 24 h after GA	
	Odds ratio (95% CI)	*P* value	Odds ratio (95% CI)	*P* value
Sugammadex (vs. neostigmine)	0.81 (0.68–0.96)	0.013	0.87 (0.77–0.98)	0.023
Female (vs. male)	2.41 (2.03–2.86)	< 0.001	2.58 (2.27–2.92)	< 0.001
Age. year	0.99 (0.98–0.99)	< 0.001	0.99 (0.98–0.99)	< 0.001
ASA physical status (vs. Class I)				
Class II	1.04 (0.86–1.26)	0.658	0.96 (0.83–1.11)	0.550
Class III	0.87 (0.63–1.20)	0.394	0.87 (0.69–1.10)	0.237
History of PONV	1.51 (1.02–2.25)	0.040	1.54 (1.14–2.09)	0.005
Body mass index, kg·m^−2^	1.00 (0.99–1.02)	0.624	0.98 (0.96–0.99)	0.003
Current smoker	0.84 (0.60–1.17)	0.311	0.68 (0.53–0.88)	0.003
Cholecystectomy or gynecological or laparoscopic surgeries	1.16 (0.98–1.37)	0.090	0.82 (0.73–0.93)	0.002
Extent of surgery (vs. minor)				
Intermediate	1.05 (0.83–1.32)	0.702	1.02 (0.83–1.24)	0.867
Major	0.83 (0.67–1.03)	0.091	1.67 (1.40–2.01)	< 0.001
Total intravenous anesthesia (vs. inhalation anesthesia)	0.33 (0.27–0.39)	< 0.001	0.49 (0.43–0.55)	< 0.001
Intraoperative steroid use	0.81 (0.69–0.95)	0.008	0.86 (0.76–0.96)	0.010
Intraoperative 5-HT_3_R antagonist use	0.83 (0.67–1.03)	0.090	0.98 (0.83–1.15)	0.768
Duration of anesthesia, hour	1.20 (1.16–1.25)	< 0.001	1.13 (1.10–1.17)	< 0.001
Postoperative opioid use	–	–	1.23 (1.05–1.44)	0.010

*CI* Confidence interval, *ASA* American society of anesthesiologists, *PONV* Postoperative nausea and vomiting, 5-HT_3_R, 5-hydroxytryptamine receptor.

## Discussion

This study found a significant association between sugammadex use and lower overall occurrence of PONV. Additionally, there was no significant interaction between sugammadex use and type of general anesthesia with respect to overall PONV occurrence.

Our findings are consistent with those of previous studies in which sugammadex was more effective than neostigmine in reducing PONV in the immediate postoperative period^5,10,11^. In the meta-analysis for which the recommendation for sugammadex was based on, the observation period for PONV in the six studies included was the immediate postoperative phase^5^. Similar results were found in a retrospective study in which sugammadex was more effective than neostigmine in reducing PONV in the recovery room^10^. However, we also showed that the significant association between sugammadex and PONV remained during the early postoperative period but disappeared during the delayed period. Previous studies reported conflicting results regarding the effect of sugammadex on PONV during the first 24 h postoperatively. An RCT of patients undergoing extremity surgery found a significant negative association between sugammadex use and PONV upon arrival in the recovery room, but not at subsequent time points^11^. Conversely, a large multicenter retrospective study reported sugammadex to be significantly associated with a decrease in PONV during the first 24 h postoperatively^6^.

However, considering the low rate of propofol-based TIVA and prophylactic steroid use, the reported PONV occurrence rate was likely underestimated. Although we also used retrospective data, the incidence of PONV in our study was similar to the recently reported early PONV incidence in a hospital in Sweden^12^ and data from our acute pain service team^13^. Regression analysis also identified previously well-known predictors for PONV, supporting the reliability of our data. Additionally, considering the increasing use of sugammadex in our institution^14^, we only included patients from 2020 to minimize the effect of possible changes in perioperative management during the study period. A recent meta-analysis reported that compared with neostigmine, sugammadex was significantly associated with a reduction in PONV^15^. However, the PONV observation periods in the 17 included studies varied considerably, and we investigated the association between sugammadex and PONV during different time windows. Future research in the effect of sugammadex on PONV would need to consider changes over time.

The idea that sugammadex use could be associated with a decrease in PONV is based on evidence regarding the effect of neostigmine on PONV; however, this is controversial^8,16^. Although anticholinesterase has been reported to contribute to PONV through several central and peripheral mechanisms^17,18^, a recent meta-analysis found no association between neostigmine and PONV occurrence rate during the first 24 h postoperatively^8^. The low-dose neostigmine (20–40 mcg.kg^−1^) used in our institution would also have affected our inconclusive results^16^. However, in our analyses, sugammadex was significantly associated with a lower early PONV rate than was neostigmine and glycopyrrolate, possibly because of the use of glycopyrrolate. Unlike atropine, glycopyrrolate does not have antiemetic properties as it cannot cross the blood–brain barrier^19^; therefore, glycopyrrolate could not offset the emetogenic effect of neostigmine. Furthermore, neostigmine has a short effect duration (20–30 min)^20^, well below 24 h postoperatively.

The meta-analysis used as a reference in the recent PONV guidelines^4^ also assessed the effect of sugammadex on PONV according to the type of general anesthesia^5^; however, the results were inconclusive because the TIVA subgroup included only one study^5^. We also investigated the effect of sugammadex on PONV according to the type of general anesthesia and found no significant interaction between sugammadex use and type of general anesthesia with respect to the primary and secondary outcomes. Therefore, the power to reveal a significant effect of sugammadex use on PONV may vary depending on the difference in PONV occurrence according to the type of general anesthesia; however, its effect on PONV may not vary depending on the type of general anesthesia.

Our study had several limitations. First, regarding limitations inherent to the study’s retrospective design, unmeasured or unknown covariates, including a history of motion sickness, intraoperative hypotension, and fluid administration, might have biased our results. However, the effect of sugammadex on PONV is an unintended outcome that was not considered during drug determination. Therefore, our observational study design with large sample size was appropriate, given its small effect size^21^. Second, the findings have limited generalizability because the study is conducted at a single tertiary university hospital. Differences in perioperative management could affect the PONV occurrence rate. Third, the number of antiemetic agents administered and type of general anesthesia were determined by the attending anesthesiologists rather than the predicted risk of PONV. Especially, 5-HT_3_R antagonists were not used in patients at low risk of PONV in our hospitals, and the regression analysis found no association between 5-HT_3_R antagonists and overall PONV. Fourth, because we could not investigate post-discharge nausea and vomiting owing to the retrospective study design, several patients discharged within 24 h postoperatively were excluded from the analysis, possibly causing selection bias. However, given that the association between sugammadex use and PONV was significant only in the early postoperative period, the selection bias might have been insignificant by the time period of our primary outcome. Lastly, because there was no cost-effectiveness analysis, our results could not justify the use of sugammadex to reduce PONV. In a worldwide survey, concern over the cost of sugammadex was reported as the primary barrier to its use^22^. A recent cost analysis study opposed its routine use to reduce only PONV^23^. Further randomized trials are required to investigate the effect of sugammadex use on PONV during the first 24 h postoperatively.

In conclusion, sugammadex use is significantly associated with a decrease in PONV during the first 24 h after general anesthesia, possibly because of a decrease in PONV during the early postoperative period rather than the delayed period. Additionally, there is no significant interaction between sugammadex use and type of general anesthesia with respect to overall PONV occurrence, suggesting that the association between sugammadex use and PONV does not vary according to the type of general anesthesia. However, our retrospective study design precludes a firm conclusion regarding the effect of sugammadex on PONV after general anesthesia.

## Methods

### Study design and population

This retrospective observational study was approved by the Institutional Review Board of Seoul National University Hospital on July 8, 2021 (Approval No. 2107–014–1233) and was conducted according to the principles of the Declaration of Helsinki and the Strengthening the Reporting of Observational Studies in Epidemiology (STROBE) statement^24^. The requirement for informed consent was waived owing to the retrospective design of the study.

This study included adult patients (≥ 18 years) who underwent elective surgery under general anesthesia in 2020. The exclusion criteria were as follows: 1) reoperation within 24 h after anesthesia; 2) discharge within 24 h after anesthesia; 3) transfer to the intensive care unit after anesthesia; 4) no reversal agent; 5) administration of cisatracurium for NMB; 6) administration of both sugammadex and neostigmine; 7) use of inhalational anesthetic and TIVA during the same surgery; 8) ASA physical status score ≥ 4; and 9) missing covariate values for the propensity score calculations. Only the first surgery was included if the same patient underwent more than one surgery under general anesthesia during 2020.

### Anesthetic management

The choice for the specific anesthesia method for each patient was decided by the attending anesthesiologist, but this was according to the institutional protocol for general anesthesia. Briefly, patients were anesthetized with either inhalation anesthetics or propofol-based TIVA. Sevoflurane or desflurane was used as the inhalation anesthetic. Nitrous oxide was not used during the study period at our institution. TIVA was performed using target-controlled propofol and remifentanil infusion. NMB was achieved to facilitate intubation and maintained using intravenous rocuronium administration. Neuromuscular monitoring was routinely performed throughout the anesthetic period using an acceleromyography device (Intellivue NMT module, Philips Healthcare, Amsterdam, Netherlands). During anesthesia, 5-hydroxytryptamine receptor (5-HT_3_R) antagonist (0.3 mg ramosetron or 0.075 mg palonosetron) and/or 5 mg dexamethasone was/were administered intravenously for PONV prophylaxis. Patients received 2–4 mg.kg^−1^ sugammadex or 20–40 mcg.kg^−1^ neostigmine and 0.4 mg glycopyrrolate intravenously to reverse NMB at the end of the surgery based on the Food and Drug Authority recommendation^25,26^. Pyridostigmine was not used as a reversal agent during the study period. The type and dosage of the NMB reversal agent were determined based on the measured depth of NMB at the end of the surgery, as well as the individual preferences of the attending anesthesiologists. The patients were extubated following neuromuscular recovery and transferred to the post-anesthesia care unit. Rescue antiemetics, such as 5-HT_3_R antagonists or metoclopramides, were administered at the attending physician’s discretion.

### Study outcomes, study groups, and data collection

The primary outcome was PONV during the first 24 h postoperatively (overall PONV). The secondary outcomes were PONV during the first 0–2 h and 2–24 h postoperatively, defined as the early and the delayed postoperative period, respectively^27^, and antiemetic use during the first 24 h postoperatively. Nurses in the post-anesthesia care unit and general wards in our institution regularly evaluated and recorded postoperative nausea and vomiting in a binary form (yes/no). The exposure groups were determined according to whether the patient received sugammadex or neostigmine as a reversal agent.

Data on demographic characteristics, medical history, and perioperative variables including PONV occurrence and antiemetic use were retrieved from electronic medical records using the Seoul National University Hospital Patients Research Environment system. PONV data were extracted from the nursing records. The following covariates previously known to influence PONV incidence were analyzed: age, sex, body mass index, current smoking status, ASA physical status, history of PONV, cholecystectomy or gynecological or laparoscopic surgery^28^, extent of surgery (minor, intermediate, or major), type of general anesthesia (TIVA or inhalation anesthesia), use of intraoperative steroids, 5-HT_3_R antagonists, or opioids, duration of anesthesia (h), and opioid use during the first 24 h postoperatively. Extent of surgery was classified according to the authors’ judgment based on a previous study (Supplemental Table 1)^29^. Postoperative opioid use included intravenous patient-controlled analgesia with opioids and rescue opioids.

### Statistical analysis

The language and environment for statistical computing R (ver. 4.0.0; R Foundation for Statistical Computing, Vienna, Austria) was used for all statistical analyses. Continuous data were presented as the means (standard deviation [SD]) or medians (interquartile range [IQR]) based on the outcome. The normality of their distribution was determined using the quantile–quantile plot and Shapiro–Wilk test. Meanwhile, categorical data were presented as numbers (%). Missing-value imputation was not performed. The following analyses were the main statistical approaches used to evaluate the association between the type of reversal agent and PONV occurrence. First, we investigated the association between the reversal agent (sugammadex vs. neostigmine) and the primary and secondary outcomes using logistic regression with adjustment for confounding variables using stabilized inverse probability of treatment weighting (sIPTW)^30^.

The propensity score for each patient was estimated in a logistic regression model predicting sugammadex use (vs. neostigmine) as a function of the following variables: age, sex, body mass index, current smoking status, ASA physical status score, history of PONV, extent of surgery, cholecystectomy or gynecological or laparoscopic surgery, type of general anesthesia, use of intraoperative steroids, 5-HT_3_R antagonists, or opioids, and duration of anesthesia. Given the lack of evidence on the association between PONV occurrence and type of volatile agent^31,32^, we integrated desflurane and sevoflurane anesthesia into inhalation anesthesia. The weights were calculated as 1/(probability of sugammadex use) for patients who received sugammadex and as 1/(1—probability of sugammadex use) for patients who received neostigmine. The weights were stabilized by multiplying the proportion of patients who received sugammadex or neostigmine, respectively.

Patients with a probability value of 0 or 1 for receiving sugammadex were excluded based on the positivity assumption. Additionally, extreme weights smaller than the 1st percentile or larger than the 99th percentile were replaced with the 1st or 99th percentile value, respectively^29^. The between-group variable balance before and after sIPTW was evaluated according to the SMD, with an SMD < 0.10 for all covariates considered to indicate that the groups were well balanced. An SMD < 0.15 was also considered acceptable. Subsequently, a binary logistic regression was performed to investigate the association between sugammadex use and the primary and secondary outcomes before and after performing sIPTW. The significance level for the secondary outcomes was adjusted for multiple comparisons using the Bonferroni correction (*P* < 0.05/3 = 0.017).

Second, multivariable binary logistic regression analyses for the occurrence of early and overall PONV were used for sensitivity analysis with the following variables included: sugammadex use, sex, age, ASA physical status, history of PONV, body mass index, current smoking status, cholecystectomy or gynecological or laparoscopic surgery, extent of surgery, type of general anesthesia, use of intraoperative steroids or 5-HT_3_R antagonists, and duration of anesthesia. Postoperative opioid use, which could not be included in the sIPTW, was additionally included in the regression analysis for the occurrence of overall PONV. The variance inflation factor (VIF) was used to evaluate multicollinearity between the variables included in the logistic regression analyses. Univariable regression analyses were not performed to select variables for the multivariable analysis model.

Third, an interaction analysis was also performed to investigate whether the type of general anesthesia influenced the association between sugammadex use and PONV occurrence. The covariates included in the aforementioned multivariable logistic regression analysis were adjusted. Owing to the retrospective design of the study, a priori power calculation was not performed. In our institution, there were changes in the regimen of intravenous patient-controlled analgesia^13^ and intraoperative non-opioid analgesics were introduced in 2019. We determined that these changes could affect the PONV occurrence rate and thus decided to only include patients who underwent surgery in 2020. With the PONV occurrence rate of patients who received neostigmine assumed to be 18%, based on our acute pain service team’s data^13^, the group sample sizes of 5918 patients for sugammadex and 4994 patients for neostigmine could achieve 90% power to reveal a difference in proportion of—2.3% between the groups.

## Supplementary Information

Supplementary Information.

## Data Availability

The datasets used and/or analyzed during the current study are available from the corresponding author upon reasonable request and with permission of the Institutional Review Board of the Seoul National University Hospital.

## References

[1] Kooij FO, Vos N, Siebenga P, Klok T, Hollmann MW, Kal JE (2012). Automated reminders decrease postoperative nausea and vomiting incidence in a general surgical population. Br. J Anaesth..

[2] Macario A, Weinger M, Truong P, Lee M (1999). Which clinical anesthesia outcomes are both common and important to avoid? The perspective of a panel of expert anesthesiologists. Anesth. Analg..

[3] Hirsch J (1994). Impact of postoperative nausea and vomiting in the surgical setting. Anaesthesia.

[4] Gan TJ (2020). Fourth consensus guidelines for the management of postoperative nausea and vomiting. Anesth. Analg..

[5] Hristovska A-M, Duch P, Allingstrup M, Afshari A (2017). Efficacy and safety of sugammadex versus neostigmine in reversing neuromuscular blockade in adults. Cochrane Database Syst. Rev..

[6] Kim JH (2020). Comparison of the effects of sugammadex, neostigmine, and pyridostigmine on postoperative nausea and vomiting: A propensity matched study of five hospitals. J. Clin. Med..

[7] Apfel CC, Roewer N, Korttila K (2002). How to study postoperative nausea and vomiting. Acta Anaesthesiol. Scand..

[8] Cheng CR, Sessler DI, Apfel CC (2005). Does neostigmine administration produce a clinically important increase in postoperative nausea and vomiting?. Anesth. Analg..

[9] Schaller SJ, Fink H, Ulm K, Blobner M (2010). Sugammadex and neostigmine dose-finding study for reversal of shallow residual neuromuscular block. Anesthesiology.

[10] Ledowski T (2014). Retrospective investigation of postoperative outcome after reversal of residual neuromuscular blockade: Sugammadex, neostigmine or no reversal. Eur. J. Anaesthesiol..

[11] Koyuncu O (2015). Comparison of sugammadex and conventional reversal on postoperative nausea and vomiting: A randomized, blinded trial. J. Clin. Anesth..

[12] Johansson E, Hultin M, Myrberg T, Walldén J (2021). Early post-operative nausea and vomiting: A retrospective observational study of 2030 patients. Acta Anaesthesiol. Scand..

[13] Jung H (2020). Effect of fentanyl-based intravenous patient-controlled analgesia with and without basal infusion on postoperative opioid consumption and opioid-related side effects: A retrospective cohort study. J. Pain. Res..

[14] Ju JW, Kim N, Yang SM, Kim WH, Lee HJ (2021). Estimated incidence of sugammadex-induced anaphylaxis using the Korea adverse event reporting system database. J. Clin. Med..

[15] Hurford WE, Eckman MH, Welge JA (2020). Data and meta-analysis for choosing sugammadex or neostigmine for routine reversal of rocuronium block in adult patients. Data Br..

[16] Tramèr MR, Fuchs-Buder T (1999). Omitting antagonism of neuromuscular block: Effect on postoperative nausea and vomiting and risk of residual paralysis. A systematic review. Br. J. Anaesth..

[17] Hood DD, Eisenach JC, Tuttle R (1995). Phase I safety assessment of intrathecal neostigmine methylsulfate in humans. Anesthesiology.

[18] Paech MJ, Kaye R, Baber C, Nathan EA (2018). Recovery characteristics of patients receiving either sugammadex or neostigmine and glycopyrrolate for reversal of neuromuscular block: A randomised controlled trial. Anaesthesia.

[19] Fuchs-Buder T, Mencke T (2001). Use of reversal agents in day care procedures (with special reference to postoperative nausea and vomiting). Eur.J Anaesthesiol. Suppl..

[20] Priya Nair V, Hunter JM (2004). Anticholinesterases and anticholinergic drugs. Contin. Educ. Anaesthesia Crit. Care Pain.

[21] Bosdriesz JR (2020). Evidence-based medicine-When observational studies are better than randomized controlled trials. Nephrology (Carlton)..

[22] O’Reilly-Shah VN, Wolf FA, Jabaley CS, Lynde GC (2017). Using a worldwide in-app survey to explore sugammadex usage patterns: A prospective observational study. Br. J. Anaesth..

[23] Hurford WE, Welge JA, Eckman MH (2020). Sugammadex versus neostigmine for routine reversal of rocuronium block in adult patients: A cost analysis. J. Clin. Anesth..

[24] Von Elm E (2007). The strengthening the reporting of observational studies in epidemiology (STROBE) statement: Guidelines for reporting observational studies. Ann. Intern. Med..

[25] US Food and Drug Administration. Neostigmine methylsulfate injection, for intravenous use initial. https://www.accessdata.fda.gov/drugsatfda_docs/label/2021/203629s003lbl.pdf (2020).

[26] US Food and Drug Administration. BRIDION^®^ (sugammadex) Injection, for intravenous use. https://www.accessdata.fda.gov/drugsatfda_docs/label/2015/022225lbl.pdf (2015).

[27] Apfel CC (2002). Volatile anaesthetics may be the main cause of early but not delayed postoperative vomiting: A randomized controlled trial of factorial design. Br. J. Anaesth..

[28] Apfel CC (2012). Evidence-based analysis of risk factors for postoperative nausea and vomiting. Br. J. Anaesth..

[29] Myles PS (2016). Minimal clinically important difference for three quality of recovery scales. Anesthesiology.

[30] Schulte PJ, Mascha EJ (2018). Propensity score methods: Theory and practice for anesthesia research. Anesth. Analg..

[31] Macario A, Dexter F, Lubarsky D (2005). Meta-analysis of trials comparing postoperative recovery after anesthesia with sevoflurane or desflurane. Am. J. Heal Pharm..

[32] Wallenborn J (2007). The impact of isoflurane, desflurane, or sevoflurane on the frequency and severity of postoperative nausea and vomiting after lumbar disc surgery. J. Clin. Anesth..

