# Supplementary material for: Effects of sugammadex versus neostigmine on postoperative nausea and vomiting after general anesthesia in adult patients:a single-center retrospective study
Source: Sci Rep. 2023 Apr 3;13:5422. doi: 10.1038/s41598-023-32730-1 (PMC10070499; doi:10.1038/s41598-023-32730-1)
Supplement: Supplementary file 1 — Supplementary Information. [file 41598_2023_32730_MOESM1_ESM.docx]

**Supplementary Table 1.** Extent of surgery classification

| **Minor surgery** |
| --- |
| Lumpectomy, hernia repair, arthroscopy, simple biopsy, eye surgery, ear surgery, nasal surgery. |
| **Intermediate surgery** |
| Cholecystectomy, appendectomy, mastectomy, thyroidectomy, transurethral resection of prostate or bladder, cystoscopy, cesarean section, tonsillectomy and adenoidectomy, tracheostomy |
| **Major surgery** |
| Any laparotomy , bowel resection, hepatobiliary surgery other than cholecystectomy, robotic or laparoscopic urogenital surgery, peripheral vascular procedure or major amputation, any aortic procedure, pancreatic or liver resection, esophagectomy, flap surgery, cardiothoracic surgery, brain surgery, spine surgery |
